# Deletion of the *HLA‐B* Gene in One of the Inherited Haplotypes in a Northern European Family

**DOI:** 10.1111/tan.70234

**Published:** 2025-05-09

**Authors:** Frank Grünebach, Tobias B. Haack, Michaela Döring, Britta Merz, Antje Petz, Christine Bauer, Martina Storz, Peter Lang, Reinhild Klein

**Affiliations:** ^1^ Department of Hematology, Oncology, Clinical Immunology, and Rheumatology, Internal Medicine II University of Tübingen Tübingen Germany; ^2^ Institute of Medical Genetics and Applied Genomics, University of Tübingen Tübingen Germany; ^3^ Department of Pediatric Hematology and Oncology University Children's Hospital, University of Tübingen Tübingen Germany

**Keywords:** gene deletion, haplotypes, histocompatibility testing, *HLA‐B* gene

## Abstract

Targeted next generation sequencing‐based *HLA* typing of a 17‐year‐old female transplant patient showed homozygosity for the *HLA‐B* allele. The segregation analysis of HLA haplotypes of family members only allowed the conclusion that the B‐allele was deleted in the haplotype inherited from the father and accordingly paternal grandfather, resulting in false homozygous genotyping. The subsequent whole‐genome sequencing of the patient and her father confirmed an approximately 85 kb deletion at 6p21.33 from the 5′ end of the *HLA‐B* to the 3′ end of the *HLA‐C* gene extending telomeric to *HLA‐C*.

A 17‐year‐old Northern European female stem cell transplant patient with myelodysplastic syndrome was typed by high‐resolution next generation sequencing (NGS) with the One Lambda AllType NGS 11‐Loci workflow (One Lambda/Thermo Fisher Scientific/BmT GmbH, Meerbusch‐Osterath, Germany) in combination with the semiconductor‐based Ion GeneStudio S5 sequencer (One Lambda/Thermo Fisher Scientific) according to the manufacturer's instructions. Briefly, after written informed consent, DNA was extracted from blood samples with the QIAamp DNA Mini Kit (Qiagen, Hilden, Germany). *HLA‐A, ‐B, ‐C, ‐DRB1, ‐DRB3/4/5, ‐DQA1, ‐DQB1, ‐DPA1* and ‐*DPB1* genes were amplified in a multiplex polymerase chain reaction (PCR). After amplicon fragmentation and adaptor ligation, template and sequencing preparation were conducted automatically on an Ion Chef system (Thermo Fisher Scientific). Template preparation comprised the individual clonal amplification of the library fragments present in the library pool across the surface of Ion Sphere Particles (ISPs) through isothermal amplification. The procedure was completed with loading the library pool onto an Ion 530 Chip and placing the loaded chip on the sequencing instrument. The Ion 530 Chip electronically detects changes in pH after polymerase‐driven base incorporation [1]. The Binary Alignment/Map (BAM) output files obtained were analysed with the TypeStream Visual NGS Analysis Software 3.0 (One Lambda/Thermo Fisher Scientific) which aligned unmapped barcode sequences to the IPD‐IMGT/HLA reference database [2] to generate individual genotype assignments.

The HLA class I typing obtained was homozygous for A*02:01:01:01 and apparently B*18:01:01:02 (Figure 1: Female transplant patient, haplotype ‘a’).

**FIGURE 1:**
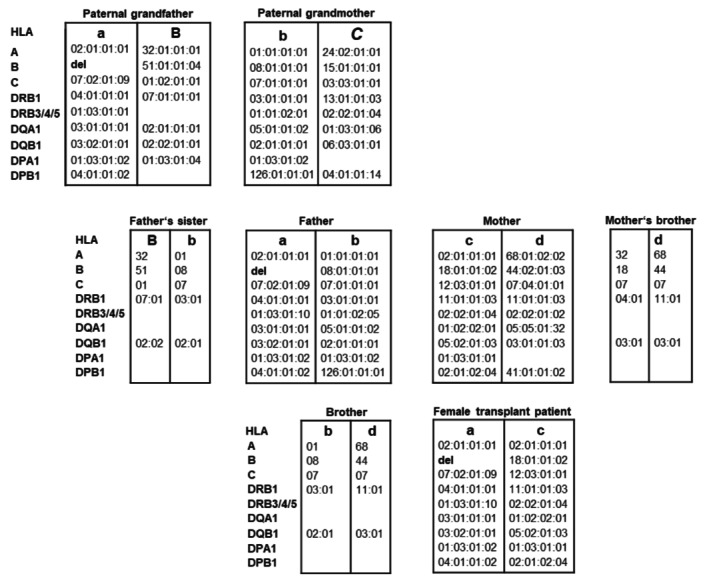
Pedigree displaying the presence of a B‐deleted haplotype in a caucasoid family. A female stem cell transplant patient, her parents and paternal grandparents were typed by high‐resolution next generation sequencing (NGS). Her brother, father's sister and mother's brother were typed at low resolution level utilising the sequence‐specific oligonucleotide (SSO) technology. Overall, six haplotypes could be segregated. The haplotype ‘a’ found in the transplant patient, her father and paternal grandfather has the *HLA‐B* gene deleted. Haplotypes related directly to the family of the patient are labelled with a, b, c, d; haplotypes related to the father's family with B, *C*. del, deletion.

When typing the patient's parents by NGS, the expected shared haplotype ‘a’ of the father presented identity with eight loci (*HLA‐A, ‐C, ‐DRB1, ‐DRB3/4/5, ‐DQA1, ‐DQB1, ‐DPA1* and ‐*DPB1*) but also a single mismatch in *HLA‐B*, which was homozygous (B*08:01:01:01; Figure 1: Father, haplotype ‘a’). Typing of the paternal grandfather by NGS also resulted in homozygosity for *HLA‐B* (in this case B*51:01:01:04) and haplotype ‘a’ again showed identity with eight loci but a mismatch in *HLA‐B* between him and his descendants (Figure 1: Paternal grandfather, haplotype ‘a’).

The brother, father's sister and mother's brother were typed at low resolution level utilising the sequence‐specific oligonucleotide (SSO) technology with the LABType kits in combination with the Luminex LABScan3D multiplex flow analyser (One Lambda/Thermo Fisher Scientific) according to the manufacturer instructions. The technique involves extraction of DNA followed by amplification of a particular HLA gene locus with biotinylated primers. The PCR products were hybridised to polystyrene microspheres which were coated with a different SSO and dyed internally with red and infrared fluorophores. Amplified DNA which has bound to the SSOs on the microspheres was detected by a fluorescent dye with the Luminex analyser using flow cytometric technology. The Luminex CSV output files were analysed with the HLA Fusion 4.6 analysis module based on catalogue specifications provided with the software (One Lambda/Thermo Fisher Scientific) [3].

The typing results of the patient's mother, brother, paternal grandmother and mother's brother did not show any inconsistencies in the identity and segregation of HLA haplotypes (Figure 1).

Taken together, the segregation analysis of HLA haplotypes of the patient's family members led us to the assumption that the B‐allele was deleted in haplotype ‘a’ (Figure 1) inherited from the paternal grandfather via the father to the patient, resulting in false homozygous genotyping of that locus.

In order to verify this hypothesis, genome sequencing of the patient was performed as described previously [4]. In brief, genomic DNA was extracted from whole blood using the FlexiGene DNA kit (Qiagen, Hilden, Germany) and quantified using the Qubit Fluorometer (Thermo Fisher Scientific, Dreieich, Germany). One microgram of genomic DNA was further processed using the TruSeq PCR‐Free Library Prep kit (Illumina, Berlin, Germany). Generated libraries were sequenced on a NovaSeq6000 System (Illumina) as 2x150 bp paired‐end reads to an average 41X coverage. Read mapping and variant calling was done using the in‐house developed megSAP pipeline (https://github.com/imgag/megSAP). Data analysis included a semiquantitative algorithm for copy number variant analysis (ClinCNV) confirming an approximately 85 kb deletion at 6p21.33, encompassing the entire *HLA‐B* gene and intergenic regions upstream to the 5′ end of the *HLA‐C* gene (Figure 2A, Allele 1). Within the general methodological limitations of short read sequencing to resolve low complexity regions, the exact breakpoints (NC_000006.12:g:31272486_31357576del) could be manually determined in few reads with single base resolution. Visual representation of the sequencing reads suggested an additional approximately 10 kb intergenic deletion on the other allele. Evaluation of the corresponding soft‐clipped reads indicated presence of the alternate locus chr6_GL000252v2_alt (Figure 2A, Allele 2). Figure 2B shows the corresponding Integrative Genomics Viewer (IGV) [5] screenshots of the patient.

**FIGURE 2:**
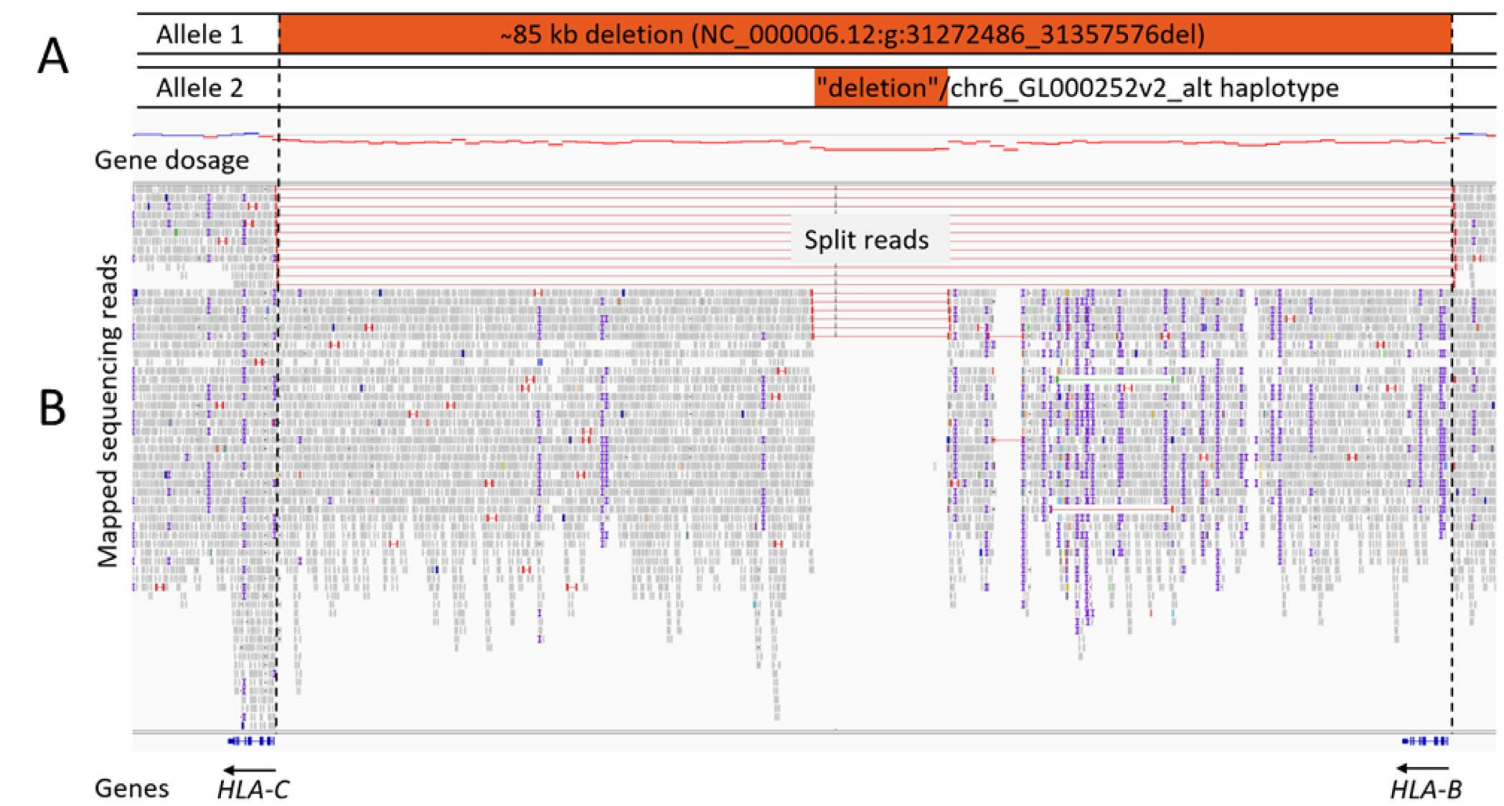
Genome sequencing confirms heterozygous *HLA‐B* deletion in a female stem cell transplant patient. (A) The upper panel shows highlighted in red a schematic representation of the two alleles with the approximately 85 kb deletion (Allele 1) and alternative haplotype with an additional approximately 10 kb intergenic deletion (Allele 2). (B) The panels below represent the results of the semiquantitative gene dosage analysis and the corresponding IGV screenshots of the patient.

A Haplotype Frequency Search in the Allele Frequency Net Database with the input of our typing results of haplotype ‘a’ for *HLA‐A, ‐C, ‐DRB1, ‐DQB1* and *‐DPB1* (Figure 1) revealed solely the haplotype A*02:01, B*07:02, C*07:02, DRB1*04:01, DQB1*03:02 and DPB1*04:01 with a frequency of 0.0828% [6]. The population of this haplotype was ‘Germany DKMS—German donors, ethnic origin: Caucasoid’ that matched the characteristics of the family analysed here. For this reason, the deleted allele is possibly B*07:02 because it is associated with a well‐conserved extended haplotype common among Europeans [7].

Deletion of an HLA gene was also reported by Lafarge. In contrast to our analysis, this was about the deletion of the *HLA‐DRB1* gene in two unrelated individuals from La Réunion Island. The author assumed, that the deleted allele is DRB1*13:01 in the African haplotype A*30:02, C*18:02, B*57:03 and DRB1*13:01 and this haplotype may be not so rare in this ethnic/geographic origin [8].

Downregulation or loss of HLA class I expression on tumour cells is a well‐known immune escape mechanism. Loss of heterozygosity (LOH), resulting from deletion of one allele at a given locus, as a somatic mutation, is the most common mechanism of HLA haplotype absence in a malignant tumour. Therefore, in hematologic malignancies, blood samples with increased blasts should not be used for HLA typing because LOH may lead to incorrect results [9, 10, 11].

In contrast, our study confirms LOH at HLA loci as germline mutations that are misinterpreted as homozygosity during routine HLA testing even in healthy subjects. However, both the occurrence of (apparent) homozygosity in the relevant population and/or underlying mechanisms and basic causes of this phenomenon remain unclear.

Determination of the frequency of deletions of one allele at the major histocompatibility complex (MHC) region could provide first insight into population genetics and also help to determine the risk of false homozygosity results in HLA genotyping. Therefore, to get a first insight, we have searched 12,914 full genomes processed via the same pipeline for deletions sharing approximate breakpoints. This led to the identification of one additional individual with the identical deletion, indicating that this constellation is very rare and likely occurs with a minor allele frequency (MAF) < 0.0001 in the central European population. However, to answer the question conclusively, systematic complex genetic studies of large study cohorts are needed which go beyond the scope of the present case report.

## Conflicts of Interest

The authors declare no conflicts of interest.

## Data Availability

The data that support the findings of this study are available from the corresponding author upon reasonable request.
